# Supplementary material for: Identification of Novel Kv1.3 Channel-Interacting Proteins Using Proximity Labelling in T-Cells
Source: Cell Physiol Biochem. Author manuscript; Available in PMC 2026 Mar 16. (PMC12989817; doi:10.33594/000000823)
Supplement: supplemental material [file NIHMS2125573-supplement-supplemental_material.zip › Supplemental Material/Supplementary Material.docx]

**Identification of novel Kv1.3 channel-interacting proteins using proximity labelling in T-cells**

Dilpreet Kour^1^#, Christine A. Bowen^2,3^#, Upasna Srivastava^1^, Hai M. Nguyen^4^, Rashmi Kumari^1^, Prateek Kumar^1^, Amanda D. Brandelli^1^, Sara Bitarafan^5,6^ Brendan R Tobin^6,7^, Levi Wood^5,6,8^, Nicholas T. Seyfried^2,3^, Heike Wulff^4^, Srikant Rangaraju^1^

**Supplementary Figures;**

**
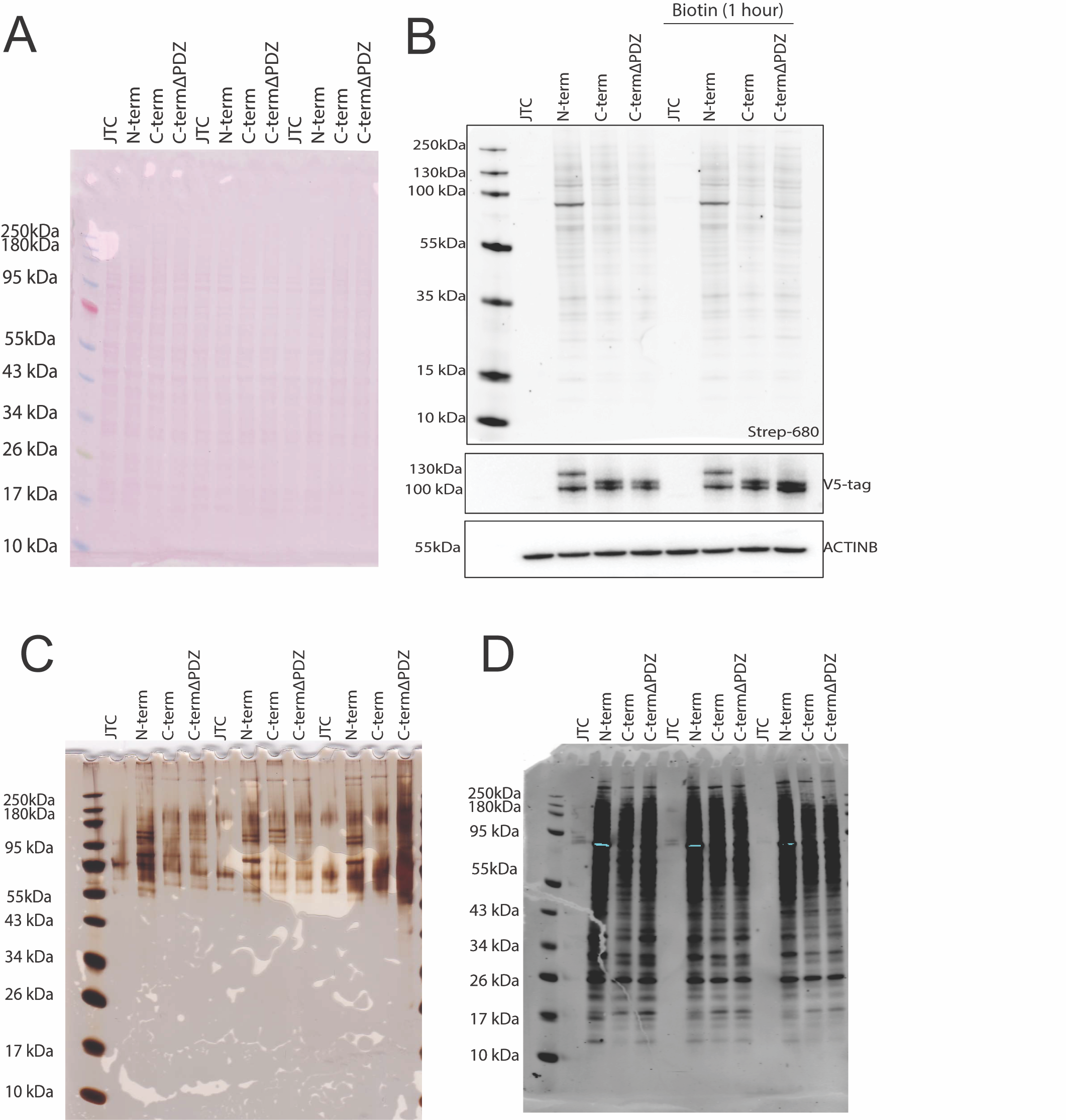
**

**Supplemental Figure 1: Verification of proteomic biotinylation and quality control assessments of streptavidin-pulldowns from Kv1.3-TurboID transduced Jurkat T-cell lysates. (A)** Ponceau staining of protein prior to probing for western blot highlights even protein loading and distribution (n=3). **(B)** 1 hour biotin pulse shows no additional effect of external biotin supplementation. V5 immunoblot represents different banding pattern in N-terminal Kv1.3-TurboID constructs, probably due to difference in post translational modifications of Kv1.3 channel in this line. **(C)** Post-Affinity purification silver stain shows successful purification of proteins present after AP (n=3). **(D)** Post-affinity purification western blot utilizing streptavidin-680 shows enrichment of biotinylated proteins following AP (n=3).


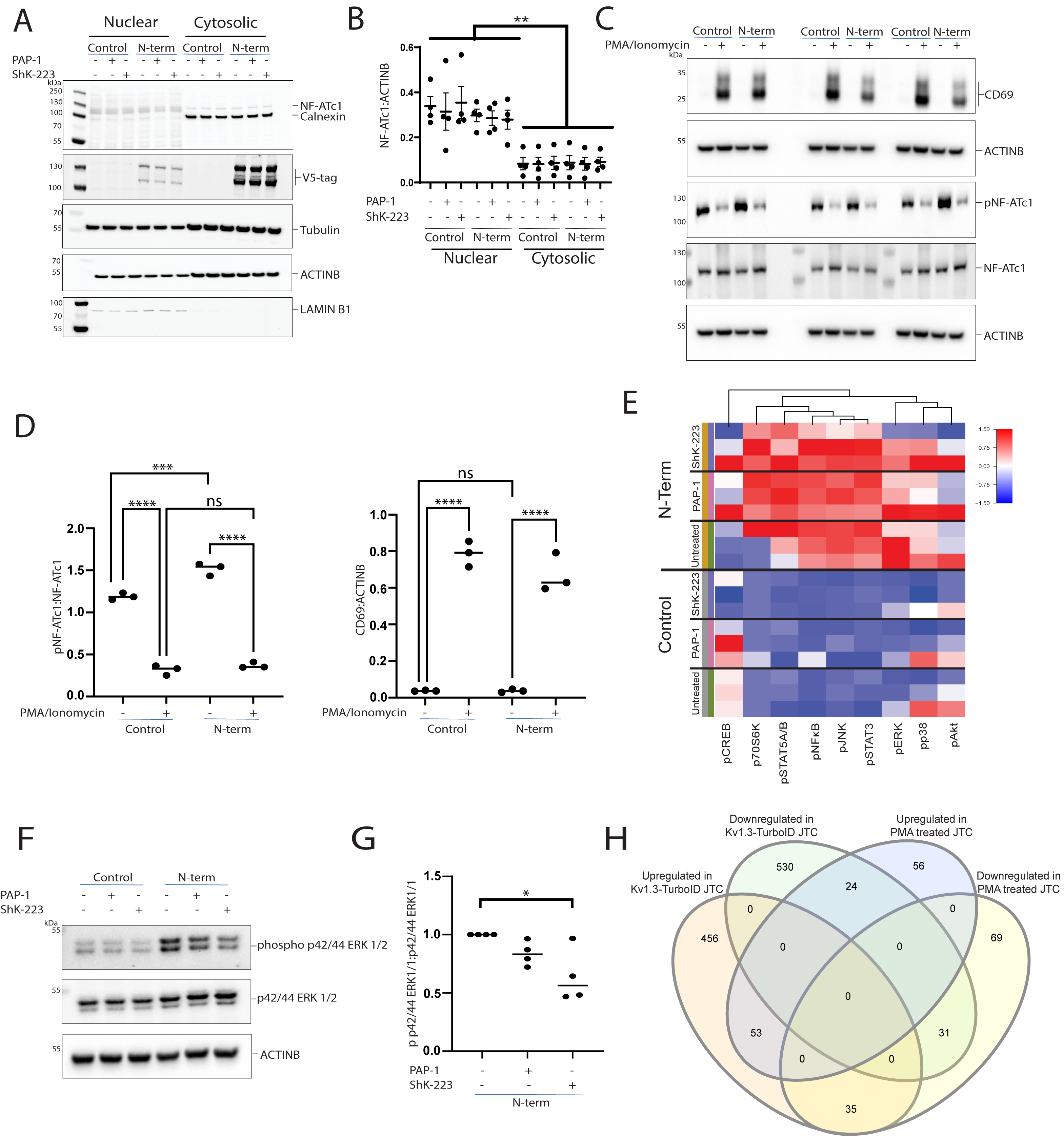


**Supplemental Figure 2: Effects of Kv1.3 channel over-expression on Jurkat T-cells properties. (A)** Western blot depicting effect of Kv1.3 channel transduction and blockade on NF-AT signaling (n=4). **(B)** Densitometric analysis of western blot given in Supp. Fig 2A. **(C)** Western blot showing effect of 20 hours PMA + Ionomycin treatment to Kv1.3 transduced and control JTC cells on CD69, and NF-AT phosphorylation (n=3). **(D)** Densitometric analysis of western blot given in Supp. Fig 2C. **(E)** Heat map showing effect of Kv1.3-TurboID constructs transduction in Jurkat T-cells and channel blockade by PAP-1 (100 nM) and ShK-223 (200 nM) on multiple signaling proteins measured by multiplex assay (n=3). **(F)** Western blot displaying impact of Kv1.3 channel blockade on ERK 1/2 phosphorylation. **(G)** Densitometric analysis of western blot given in Supp. Fig 2F. **(H)** Venn diagram analysis comparing bulk whole-cell proteomes of PMA treated JTCs (Agosto *et. al.)* with our Kv1.3-TurboID transduced JTC proteomes. The low-level of overlaps between DEPs indicate that the effects of Kv1.3 over-expression are not similar to those of JTC activation by PMA/Ionomycin. Western blot band density was analyzed in ImageJ software. Statistical analyses were done by One-way ANOVA analysis, followed by Tukey’s test. Statistical analysis of Luminex data is given in Supp. Datasheet 2. Detailed DEPs list for Figure 2H is given in Supp. Datasheet 4.


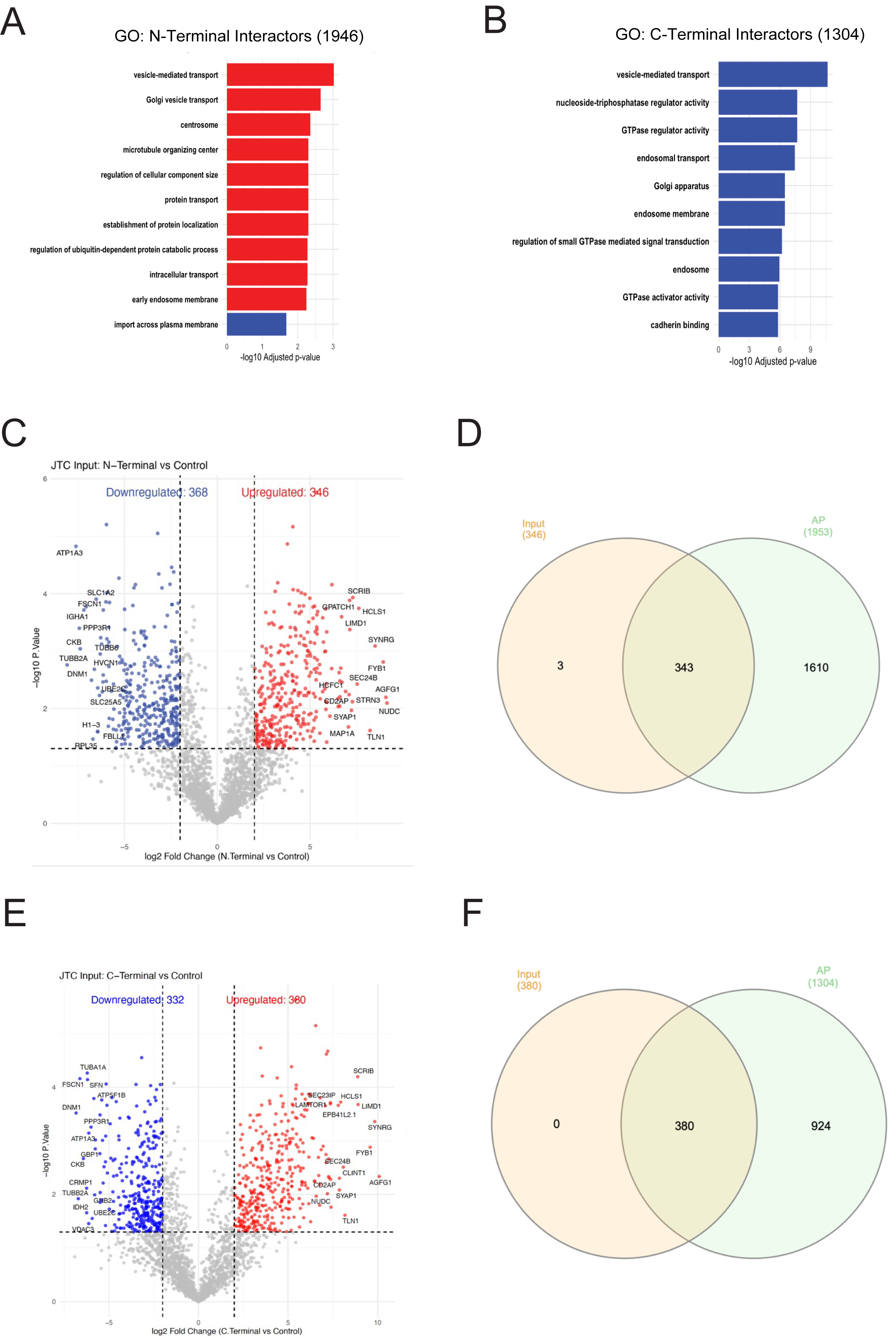


**Supplemental Figure 3: Pathway enrichment analysis of Kv1.3 terminal specific interactors. (A)** Gene Ontology (GO) term enrichment analysis of N-terminal interactors identified in Fig 3A shows Kv1.3 channel’s N-terminal role in protein trafficking and localization. **(B)** GO analysis of C-terminal interactors identified in Fig 3B displays C-terminal functioning in protein transport and regulation of GTPase activity. **(C)** DEA of N-Terminal-TurboID and Control JTC at bulk level shows 346 interactors of Kv1.3 channel’s N-terminal. **(D)** Intersection of N-terminal enriched protein at bulk and AP level reveals 1610 unique DEPs present only in the AP proteome. **(E)** DEA of C-Terminal-TurboID and Control JTC highlights 380 proteins enriched at Kv1.3 channel’s C-terminal in bulk proteome. **(F)** Intersection of C-terminal enriched protein at bulk and AP level shows 924 unique DEPs present only in the AP proteome. All related analyses are provided in Supp. Datasheet 4.


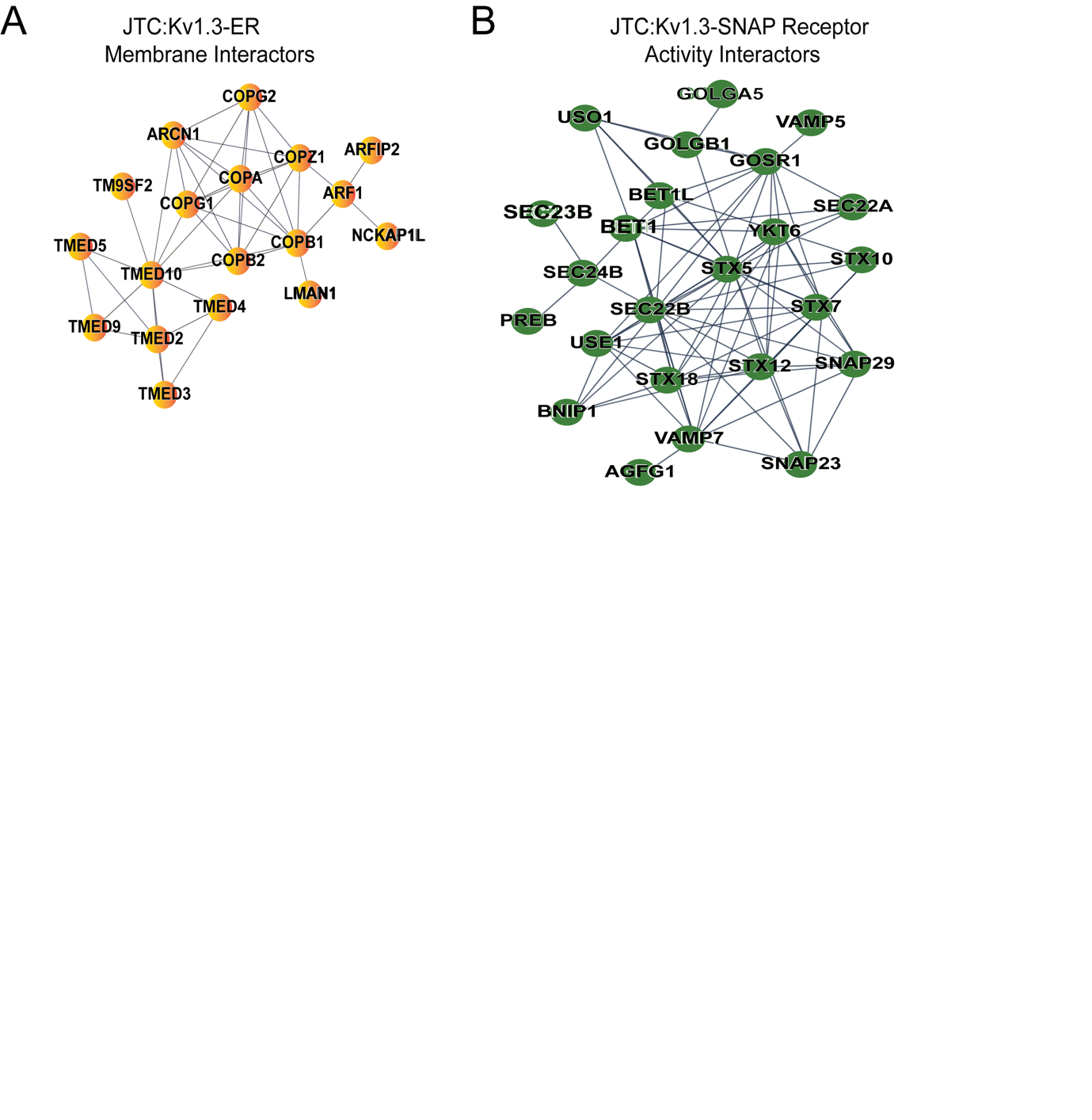


**Supplemental Figure 4: Network analysis of membrane interactors of Kv1.3 channel in Jurkat T-cells. (A)** STRING analysis of 335 membrane interactors of Kv1.3 channel identified in Fig 5A shows 18 ER membrane proteins interacting with Kv1.3 channel in JTCs. **(B)** Network analysis of Kv1.3 channel’s membrane interactors in JTCs highlights 25 proteins from SNAP receptor activity family. Details of related analyses are provided in Supp. Datasheet 6.

**Legends for Supplementary Datasheets**

**Supplementary Datasheet 1. Normalized matrix of affinity purified and Bulk proteome**

Sheet 1: Normalized matrix AP- Log2 abundance for identified proteome in streptavidin affinity-purified samples by LC-MS is given. Log2 expression values of all of the proteins were normalized to TurboID abundance.

Sheet 2: Normalized matrix Bulk- Log2 transformed abundance of identified proteome from bulk whole cell lysates input after sum intensity normalization.

**Supplementary Datasheet 2. Statistical analysis of phospho-protein signaling in Control or N-terminal TurboID-tagged Kv1.3 expressing JTCs, treated with or without Kv1.3 inhibitors.**

Adjusted p values given for each group comparison to other, for all the phospho-proteins analysed through Luminex, given in supplementary figure 2E.

**Supplementary Datasheet 3. Differentially expressed protein and GO pathway analysis of Kv1.3 associated proteins in AP and Bulk proteome in Jurkat T cells.**

Sheet 1: Differentially enriched proteins in Kv1.3-TurboID samples compared to Controls in AP proteome. The table enlists proteins significantly enriched in Kv1.3 samples.

Sheet 2: Differentially enriched proteins in Kv1.3-TurboID samples compared to Controls in Bulk proteome. The table enlists proteins significantly enriched with Kv1.3-TurboID constructs in bulk lysates.

Sheet 3: GO pathway enrichment of Kv1.3 interactors (vs. control) in AP proteome. GO term significantly enriched among Kv1.3 associated proteins are listed along with gene symbols

Sheet 4: GO pathway enrichment of Kv1.3 associated proteins (vs. control) in Bulk proteome. GO term significantly associated with Kv1.3 enrichment given along with gene symbols.

Sheet 5: Overlapping proteins in Kv1.3 enriched interactors in AP and Bulk proteome. List shows proteins increased in AP proteome upon Kv1.3-TurboID vs Control DEA and proteins which were increased as well as decreased upon Kv1.3-TurboID vs Control DEA in Bulk proteome

Sheet 6: GO enrichment analysis of Kv1.3 interactors uniquely present in AP proteome. Pathways linked to Kv1.3 interactors which are enriched specifically in AP proteome.

**Supplementary Datasheet 4. Comparison of DEPs identified in Kv1.3-TurboID cells in bulk proteome with known proteomic dataset of PMA treated Jurkat T cells.**

Sheet 1: Differentially enriched proteins in PMA treated JTC (Agosto et al. Genome Medicine 2019) listed along with DEPs identified in Kv1.3-TurboID transduced JTCs with Fold change and p values given along the proteins.

Sheet 2: Venn diagram analysis between DEPs from PMA treated JTC and Kv1.3-TurboID transduced JTCs at input level. The table enlist proteins which are unique to each dataset.

**Supplementary Datasheet 5. Differentially expressed protein and GO pathway analysis of Kv1.3 N- and C-terminal TurboID constructs in Jurkat T cells.**

Sheet 1: Differentially enriched proteins in Kv1.3 N-terminal TurboID samples compared to control in AP proteome. The table lists proteins significantly enriched in N-terminal samples, including log2 fold-change, p-values, and significance classification.

Sheet 2: Differentially enriched proteins in Kv1.3 C-terminal TurboID samples compared to control in AP proteome. Similar analysis showing proteins upregulated in the C-terminal samples.

Sheet 3: GO pathway enrichment of N-terminal interactors (vs. control). GO term significantly enriched among N-terminal interactors are listed along with gene symbols

Sheet 4: GO pathway enrichment of C-terminal interactors (vs. control). GO analysis for biological processes enriched in the C-terminal interactome are listed along with gene symbols.

Sheet 5: Overlapping and unique protein interactors between N- and C-terminal Kv1.3 (vs. Control). List includes proteins common to both terminals and those which are uniquely enriched.

Sheet 6: Differentially enriched proteins between N-terminal and C-terminal Kv1.3 constructs. Direct comparison of enrichment between the two Kv1.3 constructs, showing contrasting expression of proteins in each terminal.

Sheet 7: Overlapping proteins enriched in N-terminal samples both vs. control and vs. C-terminal. List represents strong N-terminal-specific interactors.

Sheet 8: Overlapping proteins enriched in C-terminal samples both vs. control and vs. N-terminal. List represents strong C-terminal-specific interactors.

Sheet 9: GO enrichment analysis of N-terminal specific interactors. Pathways significantly associated with proteins uniquely enriched at the N-terminus.

Sheet 10: GO enrichment analysis of C-terminal specific interactors. Pathways linked to C-terminal exclusive interactors.

Sheet 11: Overlap of N- vs. C-terminal interactors in AP-enriched versus bulk proteome data. The list shows proteins which are uniquely expressed in affinity purified (AP) proteome.

Sheet 12: GO analysis of N-terminal exclusive interactors detected only in affinity-purified proteome. Reflects biological functions of AP proteome specific interactors.

Sheet 13: Differential interactors analysis of full-length Kv1.3 C-terminal line with deleted PDZ-binding domain Kv1.3 C-terminal construct. Includes fold-change, and significance for proteins differentially recruited in presence or absence of PDZ binding domain.

Sheet 14: Differentially enriched proteins in Kv1.3 C-terminal TurboID samples compared to control in Bulk proteome (Inputs). Analysis showing proteins which are up or downregulated in C-term constructs vs Control

Sheet 15: Differentially enriched proteins in Kv1.3 N-terminal TurboID samples compared to control in Bulk proteome (Inputs). Analysis showing proteins which are up or downregulated in C-term constructs vs Control

Sheet 16: Overlapping proteins enriched in C-terminal samples in AP and bulk proteome. List shows overlapping and unique proteins enriched in C-terminal vs Control in AP and Bulk proteome

Sheet 17: Overlapping proteins enriched in N-terminal samples in AP and bulk proteome. List shows overlapping and unique proteins enriched in N-terminal vs Control in AP and Bulk proteome

**Supplementary Datasheet 6. Differentially expressed and overlapping Kv1.3 interactors in BV-2 and Jurkat T cells.**

Sheet 1: Differentially expression analysis of Kv1.3-TurboID samples and Controls in BV-2 microglial cells. The table enlists proteins significantly enriched with Kv1.3 transduction in BV-2 cells.

Sheet 2: Overlapping and unique Kv1.3 interactors in BV-2 and JTCs. List shows proteins which are uniquely enriched in Kv1.3 interactome in BV-2 and JTCs, along with proteins which are overlapping in the two cell lines.

Sheet 3: GO enrichment analysis of Kv1.3 interactors uniquely present in BV-2 cells. Pathways linked to Kv1.3 interactors which are enriched specifically in BV-2.

Sheet 4: GO enrichment analysis of Kv1.3 interactors expressed in both JTC and BV-2 cells. GO Pathways significantly linked to Kv1.3 interactors which are present in both cell types.

Sheet 5: GO enrichment analysis of Kv1.3 interactors uniquely present in JTC. Pathways linked to Kv1.3 interactors which are enriched specifically in JTC.

**Supplementary Datasheet 7. Membrane specific interactors of Kv1.3 channel in BV-2 and Jurkat T cells.**

Sheet 1: Overlap between membrane proteins and Kv1.3 enriched proteins in JTCs. Given are the list of Kv1.3 interactors in JTCs which are overlapping with list of human membrane proteins, identifying membrane interactors of Kv1.3 channel in T-cells.

Sheet 2: Overlap between membrane proteins and Kv1.3 enriched proteins in BV-2 cells. Given are the list of Kv1.3 interactors in BV-2 which are overlapping with list of human membrane proteins, identifying membrane interactors of Kv1.3 channel in microglia.

Sheet 3: STRING analysis of membrane interactors present in Kv1.3 interactome in Jurkat T cells and BV-2 cells. Different protein clusters identified in JTCs or BV-2 cells Kv1.3-membrane interactors by STRING analysis.

Sheet 4: Overlapping and unique Kv1.3 membrane interactors in BV-2 and JTCs. List shows membrane proteins which are uniquely present in Kv1.3 interactome in BV-2, JTCs, and proteins which are overlapping in the two cell lines.

Sheet 5: Overlapping and unique Kv1.3 plasma membrane interactors in BV-2 and JTCs. List shows plasma membrane proteins which are present specifically in Kv1.3 interactome in BV-2, JTCs, and plasma membrane proteins which are present in both cell lines.

**Supplementary Datasheet 8. Autoimmune disease risk genes interactors of Kv1.3 channel Jurkat T cells.**

Sheet 1: Overlap between autoimmune disease associated genes and Kv1.3 enriched proteins in JTCs. Given are the list of autoimmune disease risk genes and Kv1.3 interactors in JTCs, identifying disease associated genes that are interacting with Kv1.3 channel in JTCs.

Sheet 2: GO analysis of disease associated interactors of Kv1.3 in JTC. GO Pathways significantly linked to Kv1.3 disease interactors present in JTC.

Sheet 3: STRING analysis of autoimmune disease risk genes present in Kv1.3 interactome in Jurkat T cells. Different protein clusters identified in Kv1.3-disease risk gene interactors by STRING analysis.
